# Knowledge-Based Neuroendocrine Immunomodulation (NIM) Molecular Network Construction and Its Application

**DOI:** 10.3390/molecules23061312

**Published:** 2018-05-30

**Authors:** Tongxing Wang, Lu Han, Xiaorui Zhang, Rongrong Wu, Xiaorui Cheng, Wenxia Zhou, Yongxiang Zhang

**Affiliations:** 1 Beijing Institute of Pharmacology and Toxicology, Beijing 100850, China; wangtongxing89@126.com (T.W.); bmigroup2@163.com (L.H.); zhangx_r@126.com (X.Z.); wrr302@163.com (R.W.); zhangyx@bmi.ac.cn (Y.Z.); 2 State Key Laboratory of Toxicology and Medical Countermeasures, Beijing 100850, China

**Keywords:** neuroendocrine immunomodulation network, disease network, rheumatoid arthritis, Alzheimer’s disease, drug target, pathogenesis

## Abstract

Growing evidence shows that the neuroendocrine immunomodulation (NIM) network plays an important role in maintaining and modulating body function and the homeostasis of the internal environment. The disequilibrium of NIM in the body is closely associated with many diseases. In the present study, we first collected a core dataset of NIM signaling molecules based on our knowledge and obtained 611 NIM signaling molecules. Then, we built a NIM molecular network based on the MetaCore database and analyzed the signaling transduction characteristics of the core network. We found that the endocrine system played a pivotal role in the bridge between the nervous and immune systems and the signaling transduction between the three systems was not homogeneous. Finally, employing the forest algorithm, we identified the molecular hub playing an important role in the pathogenesis of rheumatoid arthritis (RA) and Alzheimer’s disease (AD), based on the NIM molecular network constructed by us. The results showed that *GSK3B*, *SMARCA4*, *PSMD7*, *HNF4A*, *PGR*, *RXRA*, and *ESRRA* might be the key molecules for RA, while *RARA*, *STAT3*, *STAT1*, and *PSMD14* might be the key molecules for AD. The molecular hub may be a potentially druggable target for these two complex diseases based on the literature. This study suggests that the NIM molecular network in this paper combined with the forest algorithm might provide a useful tool for predicting drug targets and understanding the pathogenesis of diseases. Therefore, the NIM molecular network and the corresponding online tool will not only enhance research on complex diseases and system biology, but also promote the communication of valuable clinical experience between modern medicine and Traditional Chinese Medicine (TCM).

## 1. Introduction

Complex diseases are characterized by multifactor induction, multisystem disorder, and multistep processes involving multiple genes [1,2]. The treatment for them lies in the equilibrium of the whole system [3,4], rather than focusing on a single target [5,6]. The theory of the biological network is suitable for understanding the pathogenesis of complex diseases [7,8,9,10]. With the development of artificial intelligence algorithms [11,12,13,14], and their in-depth application in the field of biology and medicine [15,16], a variety of networks have been constructed through either actual interactions or potential correlates among biological entities and applied to decipher pathogenesis of disease and mechanism of drug action [17,18,19,20]. However, these kinds of networks are generally too global to reflect the intrinsic mechanism of disease or drug networks. Mounting evidence indicates that the neuroendocrine immunomodulation (NIM) network plays an important role in maintaining and modulating body function balance and the homeostasis of the internal environment [21]. The NIM network incorporates information from the molecular level to the organ level of the body and mirrors the disturbance of genetic and environmental factors [22]. The disequilibrium of the NIM in the body is closely associated with many diseases [4,23,24,25,26,27]. Therefore, it is meaningful to study the pathogenesis of disease and mechanism of drug action in the context of the NIM network system, which is characterized by high pertinence. The most important thing is to map the conceptual NIM network to knowledge-based interactions between biological components (such as genes, transcripts, proteins, metabolites, etc.) from different biological levels at the micromolecule level. Some useful attempts have been made and some related studies have been carried out on this basis [28,29,30,31,32], which lay a good foundation for our research. In their research, the authors constructed a NIM molecular network mainly using text mining technology, which is based on the principle of co-occurrence. When two words co-exist in one sentence, they assume that there is a relationship between them. Obviously, the completeness and reliability of data obtained by this method need to be verified further. The direction and mechanism of mutual relationships obtained in this way are uncertain with a high false positive rate [30,31,32], but are very crucial to understanding the pathogenesis of diseases and the mechanism of drug action.

The MetaCore database (https://portal.genego.com/) is a high-precision database built from experimental results. It collects millions of interactions between biological components (such as genes, transcripts, proteins, metabolites, etc.). These interactions have regulatory relationships (activation, inhibition, and unspecified) and regulatory mechanisms (such as transcriptional regulation, phosphorylation, dephosphorylation, etc.). It is a multilayered, multidimensional background network and can be used for the construction of a high-accuracy NIM molecular network. In order to obtain a higher-accuracy NIM molecular network, we employed the well-defined molecules in the nervous, endocrine, and immune systems to construct a network based on the MetaCore database. To investigate the reliability of the NIM network, we used the forest algorithm to extract the NIM subnetworks for two complex diseases (rheumatoid arthritis (RA) and Alzheimer’s disease (AD)) and conducted the permutation test to exam the accuracy of the subnetwork. Then, to facilitate the use of NIM networks for more researchers, an online server tool was developed by us (http://www.idrug.net.cn/NIMNT/).

## 2. Results

### 2.1. The Construction of the NIM Molecular Network

**Figure 1:**
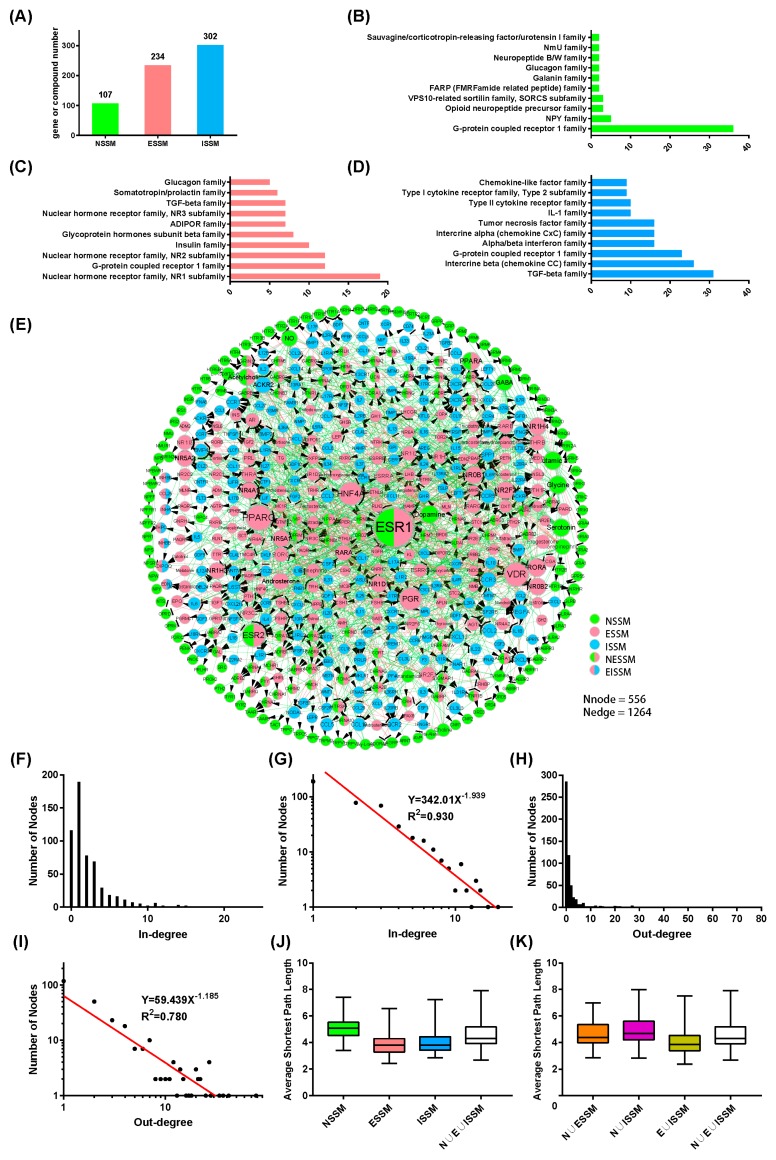
The raw data analysis for the dataset of neuroendocrine immunomodulation (NIM) signaling molecules and the core network of NIM signaling molecules. (**A**) The number distribution of the NIM signaling molecules belonging to the three systems; (**B**–**D**) The protein family distribution of NSSM, ESSM, and ISSM, Nervous system signaling molecules, NSSM; endocrine system signaling molecules, ESSM; immune system signaling molecules, ISSM; (**E**) The core network of the NIM signaling molecules. The nodes represent genes or compounds and the edges represent regulatory relationships including activation, inhibition, and unspecified. The green line with a black arrow indicates activation. The red line with small black line segments indicates inhibition. The gray line indicates that there is an unspecified interaction between the nodes. The color of the NIM signaling molecules indicates the category (NSSM is green, ESSM is pink, ISSM is blue, NESSM is green and pink, and EISSM is pink and blue). Nervous and endocrine system shared signaling molecules, NESSM; endocrine and immune system shared signaling molecules, EISSM. The size of node and font is proportional to the node degree; (**F**,**G**) The relationship of the node number and the in-degree in the core NIM molecular network; (**H**,**I**) The relationship of the node number and the out-degree in the core NIM molecular network; (**J**,**K**) The boxplot of the average shortest path length of NSSM, ESSM, ISSM, N ∪ ESSM, N ∪ ISSM, E ∪ ISSM, and N ∪ E ∪ ISSM. Nervous system union endocrine system signaling molecules, N ∪ ESSM; nervous system union immune system signaling molecules, N ∪ ISSM; endocrine system union immune system signaling molecules, E ∪ ISSM; the union of the nervous system, endocrine system, and immune system signaling molecules, N ∪ E ∪ ISSM.

According to the inclusion criteria described in Materials and Methods, we obtained 611 NIM signaling molecules including 543 genes and 68 compounds (Table S1). There are 107 (including 86 genes and 21 compounds, Tables S2 and S3), 234 (including 187 genes and 47 compounds, Tables S4 and S5), and 302 (Table S6) signaling molecules in the nervous system, endocrine system, and immune system, respectively (Figure 1A). In the dataset, the NIM signaling molecules can be divided into five categories: the nervous system signaling molecules (NSSM), the endocrine system signaling molecules (ESSM), the immune system signaling molecules (ISSM), the nervous and endocrine system shared signaling molecules (NESSM), and the endocrine and immune system shared signaling molecules (EISSM).

To find out the differences and connections in the protein family between the signaling molecules of these three systems, we analyzed the family of these protein molecules in the dataset (Figure 1B–D). The results showed that the molecules of these three systems have common and different characteristics in the classification of the protein families. For example, all of the three systems contain many of the proteins belonging to the G protein-coupled receptor family. Both the endocrine and immune systems have more proteins classified into the TGF-beta family. The nuclear hormone receptor family is the characteristic protein family for signaling molecules of the endocrine system. The Chemokine CC family and the Alpha/beta interferon family are the characteristic protein families for the signaling molecules of the immune system. According to the classification of the protein family, the signaling molecules of the three systems have relatively independent characteristics and the receptor parts of the signaling molecules have a certain similarity. This may be the molecular basis keeping the three systems independent from or connected to each other.

Although we do as much as possible to obtain signaling molecules between cells, the tendency of tissue distribution is always inevitable. We investigated the tissue specificity of the gene part in the signaling molecule dataset (Figure S3) and found that the molecules belonging to the nervous system were mainly distributed over the brain, pituitary, spinal cord, and hypothalamus; the molecules of the endocrine system were in the pituitary, pheochromocytoma, kidney, and thyroid; and the molecules of the immune system were in the blood, monocytes, spleen, and leukocytes. The tissue distribution of the signaling molecules is consistent with the physiological functions of the organs. There are some common tissues containing the signaling molecules from different systems, such as the pituitary and blood. These overlapping organs are also the physiological basis of the three major system connections.

The in-degree and out-degree distributions of the core network of the NIM signaling molecules followed p(k) ∝ k^−1.939^ and p(k) ∝ k^−1.185^, respectively (Figure 1G,I). The results suggest that the core network is a scale-free network, in which most of the nodes have small degrees, whereas a few nodes have large degrees (hub nodes) (Table 1). This property makes the network very robust.

### 2.2. Analysis of the NIM Molecular Network Characteristics

**Figure 2:**
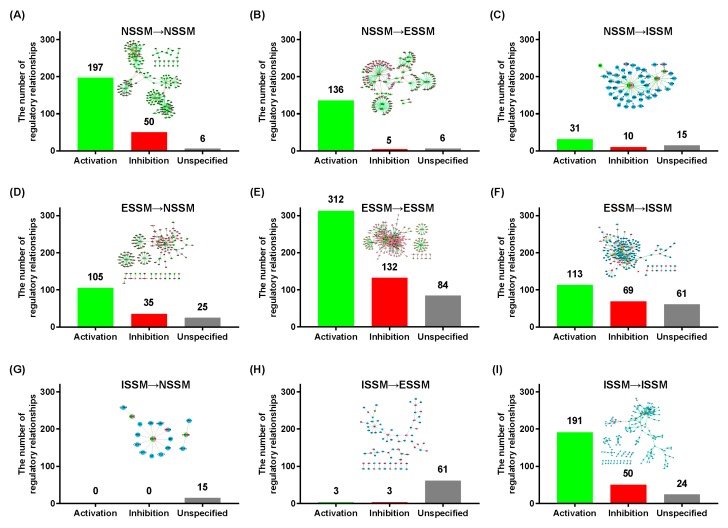
The regulatory relationships among neuroendocrine immunomodulation (NIM) signaling molecules belonging to different systems in the core molecular network. The *x* axis represents the three types of regulatory relationship (activation, inhibition, unspecified), and the *y* axis represents the number of regulatory relationships. The meaning of the network elements is the same as that shown in Figure 1. (**A**) The internal regulatory relationship of NSSM and its network form; (**B**) The regulatory relationship from NSSM to ESSM and its network form; (**C**) The regulatory relationship from NSSM to ISSM and its network form; (**D**) The regulatory relationship from ESSM to NSSM and its network form; (**E**) The internal regulatory relationship of ESSM and its network form; (**F**) The regulatory relationship from ESSM to ISSM and its network form; (**G**) The regulatory relationship from ISSM to NSSM and its network form; (**H**) The regulatory relationship from ISSM to ESSM and its network form; (**I**) The internal regulatory relationship of ISSM and its network form. Nervous system signaling molecules, NSSM; endocrine system signaling molecules, ESSM; immune system signaling molecules, ISSM.

We analyzed the signaling transmission characteristics of the core network of signaling molecules (Figure 2). As a whole, the regulatory relationship between nodes in the NIM molecular network is mainly activation, with fewer occurrences of inhibition relationships. We counted the number of regulatory relationships within any system and between any two systems. The results showed that the links between nodes within each system are more closely related (Figure 2A,E,I), and the connections between the nodes belonging to different systems are relatively few (Figure 2B–D,F–H). The molecules of the immune system rarely have a direct regulatory effect on the nervous system or endocrine system (Figure 2G,H).

Through analyzing the characteristics of the NIM molecular network, we found imbalance characteristics of signaling transduction between the three systems. The nervous system is closely related to the endocrine system (Figure 2B,D). Meanwhile, the endocrine system has a unidirectional regulatory characteristic with the immune system (Figure 2F,H). There are a few direct interactions from the immune system to the nervous system (Figure 2C,G). However, this does not mean that the immune system does not affect the nervous system, because our analysis here is only at the molecular level. At the tissue and organ levels, the nervous system and immune system have a close relationship. The immune system can modulate brain function through producing some certain cytokines in the brain, subsequently increasing the activity of the neuronal and immune systems.

We drew the regulatory network map within and between the three systems (Figure 2) and computed their topology parameters (Tables S11–S19). The regulatory network map within each system showed the differences and had its own characteristics. The network map of the nervous system presents a radiant distribution centered on chemical neurotransmitters or neuropeptides (Figure 2A). Receptors in the nervous system showed better specificity (showing one-to-one interaction). Only a small number of receptors are associated with two or more ligands. The network map of the endocrine system presents a closely connected structure centered on a few nodes such as *ESR*, *HNF4A*, and *PPARG* (Figure 2E). The molecular network of the immune system has the characteristics of the nervous system and the endocrine system at the same time (Figure 2I). They contain highly specific receptors, such as *CSF2RB*, *IL17RA*, and *CXCR2*, and some closely related structures, simultaneously.

From the regulatory network map between the nervous and endocrine systems (Figure 2B,D), we found that there are many nodes in the network belonging to the nervous and endocrine system, simultaneously, such as *ESR1*, *ESR2*, and *GABR*. Those genes played an important role in strengthening the connection between the two systems. The endocrine system has a very close relationship to the immune system (Figure 2F). *ESR1*, *ESR2*, *PGR*, *PPAGR*, *VDR*, and plenty of other receptors in the endocrine system exert a great effect on a great number of signaling molecules in the immune system. *ESR1*, *ESR2*, and *PPAGR* also played a key role in connecting the endocrine and immune systems.

The NIM molecular network, which was merely constructed using the core dataset of the signaling molecules, shows the biological network characteristics, indicating its robustness. From the signaling transduction direction among the three systems, the nervous system and the endocrine system are closely related to many common molecules. There are numerous shared receptors and other signaling molecules (Figure 2B,D), showing a mutually regulated relationship. The signaling molecules of the immune system are more connected with those in the endocrine system (Figure 2H) and less connected with those of the nervous system (Figure 2G). The direction of the signaling transduction is mostly from the endocrine system to the immune system, showing the unilateral regulation by the endocrine system of the immune system (Figure 2F,H).

To measure the distance or the degree of connecting tightness between the nodes in the core network, we took the average shortest path length as the parameter and drew the probability density curve of it for NSSM, ESSM, ISSM, N ∪ ESSM, N ∪ ISSM, E ∪ ISMM, and N ∪ E ∪ ISSM (Figure S1). The results showed that the distance between NSSM is sparser than those between ESSM and ISSM (Figures S1A and S2). The association between ESSM is most closely related to the network. The integration of any two systems can make the network more closely related (Figures S1B and S2). 

### 2.3. Case One of Applying the NIM Molecular Network: Rheumatoid Arthritis 

**Figure 3:**
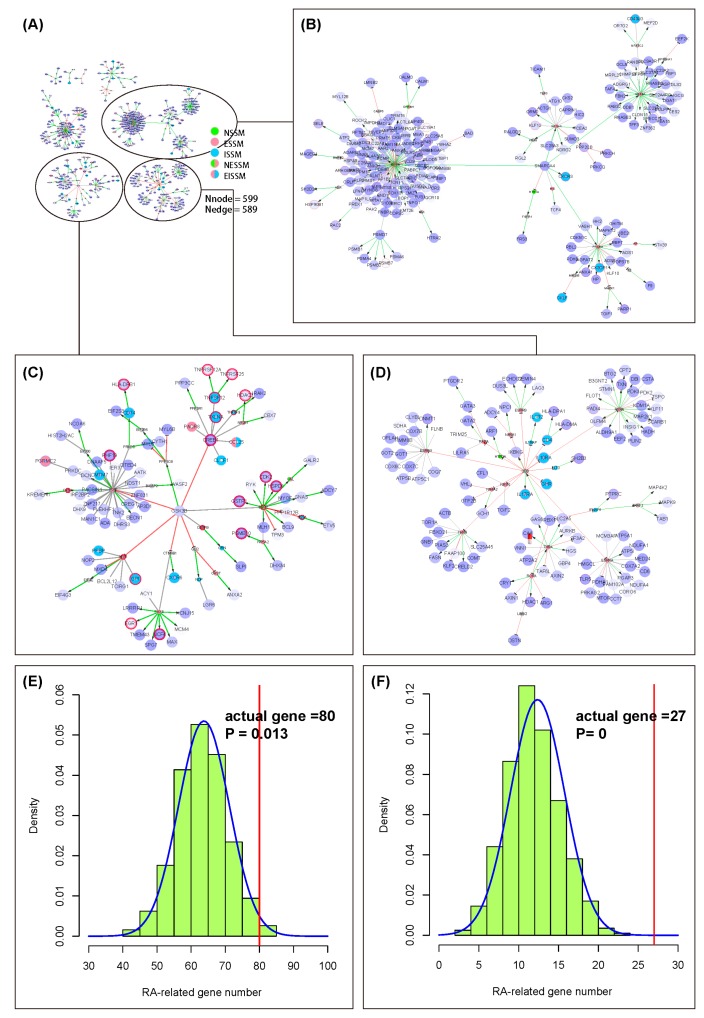
The application of the neuroendocrine immunomodulation (NIM) molecular network in the prediction of disease targets. (**A**) The overall graph of the rheumatoid arthritis (RA)-specific NIM subnetwork. The big nodes with purple color are the differentially expressed genes (DEGs); the deeper the color, the higher the fold change in the differential expression of the genes. The small nodes are the non-differentially expressed genes (non-DEGs). In addition, the nodes with a red circle are the RA-related genes. The meanings of the other network elements are the same as shown in Figure 1. (**B**,**C**) The partial enlargement network graph of (**A**); (**D**) The probability density curve of the number of RA-related genes obtained from the DEGs by permutation test; (**E**) The probability density curve of the number of RA-related genes obtained from non-DEGs by the permutation test. The RA-related genes are the biomarkers dataset of the RA download from the MetaCore database. The red vertical line is the actual number of RA-related genes in the RA-specific NIM subnetwork.

We took GSE15573 [33] in the Gene Expression Omnibus (GEO)database as the gene expression profile and analyzed it through the GEO2R tool to get differentially expressed genes (DEGs) (*p* < 0.05) (Table S20). We extracted RA-specific NIM subnetworks by taking the extended network of the NIM signaling molecules as the background. In this subnetwork, there are 599 nodes and 589 edges (Figure 3A) (Table S24). The nodes in this subnetwork were categorized into four kinds: the differentially expressed and RA-related genes (80); the non-differentially expressed and RA-related genes (27); the differentially expressed and RA-unrelated genes (421); and the non-differentially expressed and RA-unrelated genes (71). We list all the genes in the four categories in Table S22. Here, we show the top 4 hub nodes in each category (Table 2). The RA-unrelated genes, such as *GSK3B*, *SMARCA4*, *PSMD7*, *PGR*, *RXRA*, *ESRRA*, are worthy of more attention. These may be potential targets for the prevention and treatment of RA, which deserve further experimental verification.

The network graph showed that the downstream nodes of the hub non-differentially expressed genes such as *PAX8*, *ESR2*, and *PGR* often have significant changes at the transcriptional level (Figure 3C). 

In general, most researchers are concerned about the genes that are expressed differentially, and we found only 80 RA-related genes among them (Figure 3E). The permutation test was used to examine the reliability of DEGs in the subnetwork and the RA-related genes were numbered. The probability density curve followed the normal distribution with a mean of 63.76 and a standard deviation of 7.46. The outcome displayed that there was a difference in the RA-related gene number between the actual gene number and the random sampling results, but it was not very significant (*p* = 0.013) (Figure 3E). As for the non-DEG part, the permutation test results showed that the average number of RA-related genes is 12.36, which is far less than the actual gene number (27) (*p* = 0) (Figure 3F). These results indicated the credibility of the RA-specific NIM subnetwork and reminded us that only paying attention to DEGs may ignore some other important genes.

### 2.4. Case Two of Applying the NIM Molecular Network: Alzheimer’s Disease

**Figure 4:**
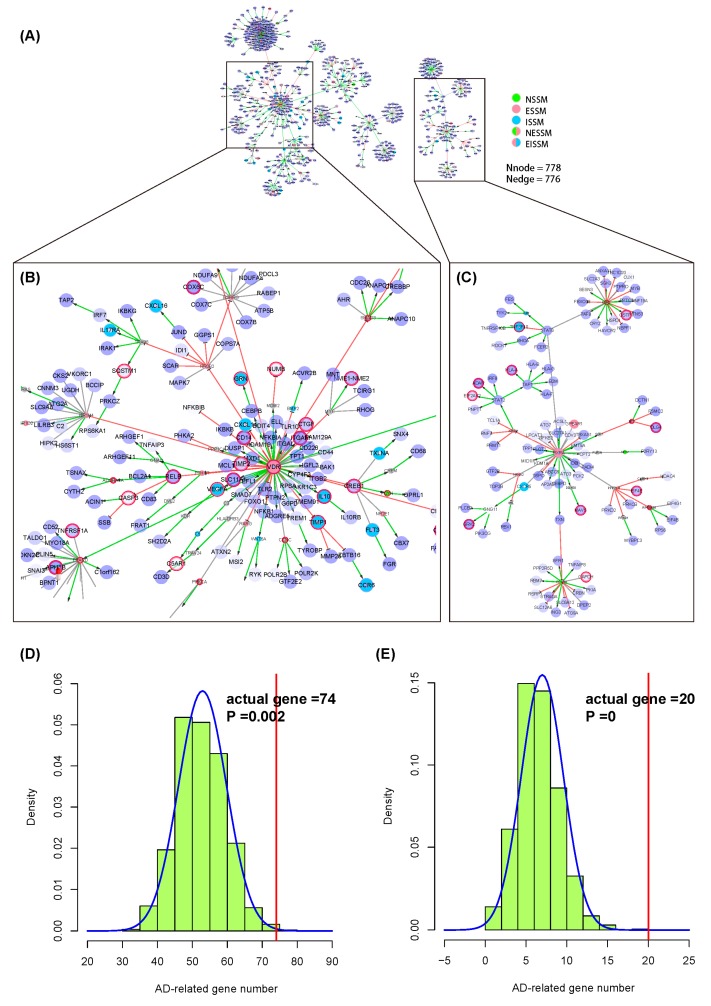
The application of the neuroendocrine immunomodulation (NIM) molecular network in the interpretation of the disease pathogenesis. (**A**) The overall graph of Alzheimer’s disease (AD)-specific NIM subnetwork. The big nodes with purple color are the DEGs. The deeper the color, the higher the fold change in the differential expression of the genes; the small nodes are the non-DEGs. In addition, the nodes with a red circle are RA-related genes. The meanings of the other network elements are the same as shown in Figure 1. (**B**,**C**) The partial enlargement network graph of (**A**); (**D**) The probability density curve of the number of AD-related genes obtained from DEGs by the permutation test; (**E**) The probability density curve of the number of AD-related genes obtained from non-DEGs by the permutation test. The AD-related genes are the biomarkers dataset of AD downloaded from the MetaCore database. The red vertical line is the actual number of AD-related genes in the AD-specific NIM subnetwork.

We took GSE63061 [34] in the GEO database as the gene expression profile of AD and screened differentially expressed genes (DEGs) (*p* < 0.05) through the GEO2R tool (Table S21). Based on the extended network of NIM signaling molecules, we extracted an AD-specific NIM subnetwork, including 778 nodes and 776 edges (Figure 4A). We also conducted a permutation test for this subnetwork as described in the first case study. The probability density curve (Figure 4D,E) showed that the number of AD-related genes was nearly 3 times higher than those obtained by random sampling, indicating that the AD-specific NIM subnetwork constructed by the NIM molecular network is reliable with a high sensitivity in detecting the disease targets. 

There are two subnetworks in the AD-specific NIM subnetwork (Table S25). The nodes *ESR1*, *VDR*, *ESRRA*, *NR0B2*, *NR1H4*, *PPARD*, *PPARG*, *SP1*, *THRB*, *ESRRA*, and *PGR* are the hub nodes in the bigger subnetwork (Figure 4B). The nodes *HNF4A*, *ESR2*, *STAT6*, *RXRA*, *PPARA*, *PPP2CB*, *STAT2*, and *NR4A1* are the hub nodes in the smaller subnetwork (Figure 4C).

## 3. Discussion

The strategy used to construct the NIM molecular network is mainly based on the technology of text mining with the drawbacks of the uncertain direction and mechanism of the mutual relationships, the low accuracy of the molecule dataset, and the high false positive rate of interactions [30,31,32]. The accuracy has been improved through the adoption of two literature mining approaches [32] or by combining literature mining with gene expression analysis [31]. However, this is unable to break through the limitations of the technology of text mining. In this study, we only chose signaling molecules with clear definitions and classifications to construct the NIM dataset, and constructed the NIM molecular network based on the MetaCore database. It is a multilayered, multidimensional background network, and can be the most suitable database for the construction of a NIM molecular network with the highest accuracy and the lowest false positive rate.

Mapping the changed molecules (such as genes, proteins, metabolites) to known or predicted interaction networks to construct a disease- or drug-specific subnetwork can help us to find the relationships between them. There are many methods that can be adapted to infer the networks, such as the maximum likelihood [35], the network alignment [36], linear programming [37], network inference from gene expression [8], the Steiner tree approach [38], electric circuits [39], network flow optimization [40], Bayesian networks [9], and network propagation [10]. Most of them employ protein–protein interactions. Moreover, there were false negatives and positives in the omics data and the interactome. These factors make it difficult to get meaningful connections and uncover previously unrecognized processes, which could provide a relatively holistic perspective of the changes that occur to a specific state of disease or drug disturbance. In our study, there are directional edges (activation, inhibition, unspecified) and multiple types of nodes (genes, proteins, metabolites, and so forth) in the NIM molecular network. The NIM molecular network constructed by us is a multilayered, multidimensional network, so the methods mentioned above are unsuitable for this task. Some studies indicated that the forest algorithm has optimum advantages in the study of mammalian cells responding to a large number of hormones, growth factors, and cytokines [41], and is used to reconstruct networks with multiple types of interactions and suited to discovering multiple pathways [41]. Therefore, we chose the forest algorithm to construct the specific NIM subnetwork of diseases, for example, RA and AD.

In the first study case, on the one hand, we found that the differentially expressed genes (such as *CREB1* [42,43], *TNFSF10* [44], *CD4* [45], and *TNFSF12* [46]), and the non-differentially expressed genes (such as *ESR1* [47], *VDR* [48], *PPARG* [49], *ESR2* [50]) are RA-related genes. On the other hand, we also found that some of the hub nodes in the network are not included in the RA-related gene dataset, such as differentially expressed genes *GSK3B*, *SMARCA4*, *PSMD7*, *TGFBR1*, and so forth, and non-differentially expressed genes *HNF4A*, *PGR*, *RXRA*, *ESRRA*, and so forth. *PGR* is associated with tumors and inflammation and the risk of RA is increased during pregnancy [51]. *GSK3B* is associated with a variety of tumors and neurodegenerative diseases [52,53,54] and the inhibitor of *GSK3B* has the effect of remission on the mouse model of RA, showing an anti-inflammatory effect [55]. Some studies have shown that cancers and neurodegenerative diseases are associated with the immune system [56,57,58]. Therefore, it may also have an important impact on the incidence of RA. Similarly, *SMARCA4* plays a key role in the development of multiple tumors [59] and its relationship with RA is also worth studying. Therefore, the hub RA-unrelated genes, such as *GSK3B*, *SMARCA4*, *PSMD7*, *HNF4A*, *PGR*, *RXRA*, and *ESRRA*, may be potential targets for the prevention and treatment of RA; this deserves more attention and further experimental verification.

Similarly, in the second study case, we found that the differentially expressed (such as *VDR* [60], *SP1* [61], *CREB1* [62], and *RELB* [63]) and the non-differentially expressed (such as *ESR1* [64], *PPARG* [65], *ESR2* [66], and *PPARD* [67]) genes are AD-related genes. Besides this, we also found that some of the hub nodes in the network are not included in the AD-related gene dataset, such as the differentially expressed genes *RXRA*, *STAT3*, *STAT1*, *PSMD14*, and so forth, and the non-differentially expressed *HNF4A*, *PGR*, *ESRRA*, *THRB*, and so forth, which are worth paying more attention to in further study. The hierarchical relationships with an explicit effect and the mechanisms between genes play an important role in the interpretation of the pathogenesis of AD as well as of other complex diseases. The downstream molecules of *VDR* were expressed abnormally. Research showed that the incidence of AD was closely related to vitamin D [68,69,70,71] and *VDR* [60,72]. For hub nodes, such as *CREB1*, *PPARD*, *ESR2*, and *SP1*, there has been evidence of associations with the onset of AD [61,66,73,74,75,76,77,78,79]. Some of the hub nodes in the network, such as *RARA*, *STAT3*, *STAT1*, and *PSMD14*, are likely to be potential genes associated with AD. For example, the *RARA* pathway in the phosphorylation of the tau protein may play a key role [80] and *RXRA* mutation is associated with AD [81]. The downstream genes regulated by *VDR*, *ESR1*, and *PGR* have abnormal expression levels, suggesting their direct influence. These genes centered on the network of diseases exert a significant impact on the downstream genes. 

In summary, prediction of the targets of complex diseases by the NIM network constructed by us was specific and sensitive. We also developed an online server tool (http://www.idrug.net.cn/NIMNT/). The gene or protein list related to the disease or drug as an input is used to extract a disease- or drug-specific NIM subnetwork easily and quickly, which can then be downloaded by a researcher. 

However, there are still shortcomings for the NIM molecular networks based on the existing knowledge in this study. There may be some new interactions among the molecules which have not been found. Therefore, our study only reflects the results of research on the NIM signaling molecules at the present stage. In later research, on the one hand, we will add the distribution information of the signaling molecules in the tissues and organs to the NIMNT, which would be very valuable for revealing the pathogenesis of diseases and the effect of drugs. On the other hand, we will conduct an experimental verification of the key factors and principal pathways predicted in the RA and AD study cases by using in vivo or in vitro experiments. The NIM network provides a bridge to connect modern medicine and traditional medicine, adding valuable clinical experience to systems biology [22]. Liuwei Dihuang decoction (LW), a classical prescription, has been used in traditional Chinese medicine (TCM) to prevent and treat various diseases with characteristic features of kidney yin deficiency. In our previous studies, we found that LW plays an integrative role in modulating the balance of the NIM network through regulating the communications and interactions between the neuroendocrine and immune systems in a bidirectional manner [82]. However, the NIM molecular network regulated by LW is unknown, and its mechanism of action at the molecular level and system angle still need to be revealed. Therefore, we will construct the NIM molecular network regulated by LW in further study in order to interpret its mechanism in treating different diseases. Considering the functional similarities between the “kidney” of traditional medicine and the NIM network of modern medicine [82], we can explore the biological foundation of “kidney” syndrome, which will promote the understanding of TCM theory. Furthermore, we can interpret the mechanism of TCM in treating the TCM syndrome, and we can find the commonalities of a variety of diseases with “kidney” syndrome at the molecular level and interpret the mechanism of homotherapy for heteropathy of LW.

## 4. Materials and Methods 

**Figure 5:**
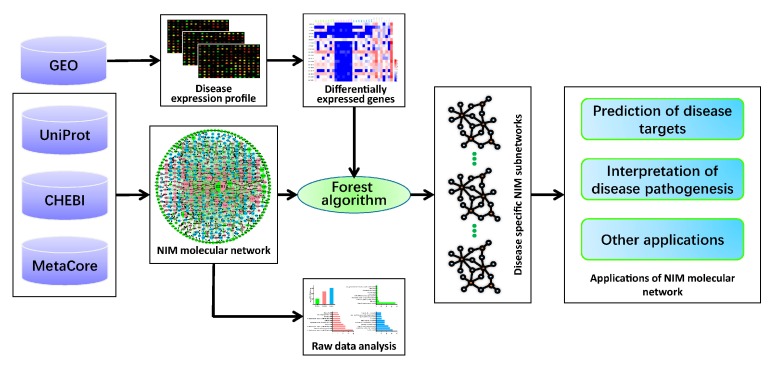
The flowchart for the construction of the NIM molecular network and its application. Firstly, we collected the NIM signaling molecules (including neurotransmitters, neuropeptides, hormones, cytokines, and their corresponding receptors) through retrieval from the UniProt and ChEBI databases. Based on the interactions in the MetaCore database, we constructed the core and the extended network of the NIM signaling molecules and conducted an analysis of the raw data and the core network. Secondly, we obtained the gene expression profile of the disease from the GEO database and screened the differentially expressed genes (DEGs). Then, we extracted the disease-specific NIM subnetworks from the extended network of NIM signaling molecules using the forest algorithm. Finally, we took the RA- and AD-specific NIM subnetworks as an example to show the application of the NIM molecular network in the prediction of the disease targets and the interpretation of the disease pathogenesis.

The flowchart for the construction of the NIM molecular network and its application is shown below (Figure 5).

### 4.1. Construction of the NIM Signaling Molecule Dataset

The NIM signaling molecules include genes and compounds. The NIM genes refer to genes categorized as neurotransmitters, neuropeptides, hormones, cytokines, and their corresponding receptors in “Gene Ontology (molecular function)” [29]. The UniProt database is a comprehensive resource for protein sequences and annotation data [83]. We used keywords and gene ontology terms (Table S1) to retrieve the NIM genes from the UniProt database. Only those reviewed as “human” in the popular organism entries were chosen as the candidate genes. Here, the NIM compounds refer to the compounds categorized as neurotransmitters, hormones under “Chemical Ontology” in the Chemical Entities of Biological Interest (ChEBI) database [84] containing a chemical ontology relating the small molecules with each other. We used the chemical ontology terms (Table S1) to retrieve the NIM compounds from the ChEBI database. The definition of hormones in the ChEBI database includes the endogenous compounds, non-endogenous compounds, and the semi-synthetic and fully synthetic analogs of such compounds. Among them, we only chose the endogenous compounds with biological role(s) as “Hormone–human metabolites” as the candidate hormones. Similarly, we chose the compounds with biological role(s) noted as “Neurotransmitter–human metabolites” as the candidate neurotransmitters. 

### 4.2. Protein Family Analysis of NIM Signaling Molecules

In order to study the distribution of the gene part of the signaling molecules in the different protein families, we used the data downloaded from the UniProt database (Tables S2, S4, and S6) to count the species of the protein family and the gene numbers contained in it.

### 4.3. Tissue Specificity Analysis of NIM Signaling Molecules

In order to study the distribution of the gene part of the signaling molecules in different tissues, we used the datasets of UP_TISSUE in the The Database for Annotation, Visualization and Integrated Discovery (DAVID) v6.8 [85] to conduct the tissue specificity analysis. Then, we counted the species of tissues and the genes numbers contained in it (Tables S7–S9). 

### 4.4. Construction and Analysis of the Network of NIM Signaling Molecules

To construct the network of the NIM signaling molecules, the interactions in the MetaCore database were taken as the background network. It is a multilayered, multidimensional background network and can be the most suitable database for the construction of a NIM molecular network with high accuracy. Firstly, the core network of the NIM signaling molecules was constructed. We imported the signaling molecule dataset into the MetaCore database and chose the direct interaction algorithm to build the core network of the NIM signaling molecules. In the core network, only NIM signaling molecules are contained. 

Considering the applicability of the NIM network, we further built the extended network of the NIM signaling molecules based on the core network. Based on the core network, the algorithm expanded by one of the interactions was chosen with upstream and downstream to simultaneously expand to obtain an upstream- and downstream-expanded network. In addition, the two-step shortest path algorithm was adopted for the core network to construct the shortest path extended network. The above two expanded networks were combined to obtain the extended NIM network of the signaling molecules (Table S28). The extended network is characterized by the backbone structure formed by the NIM core signaling molecules. It is also included in the relationships of the upstream and downstream biological entities in one step, which may affect or be affected by the NIM signaling molecules. It is a network centered on the core network of the NIM signaling molecules, which can be taken as the background network for the NIM subnetwork for extraction of diseases.

For the core network, we used the NetworkAnalyzer (release 2.7) plugin [86] for Cytoscape v3.5.1 [87] to compute the topological parameters of the core network (Table S10). Among them, the degree parameter was chosen to screen hub nodes (the nodes with a degree greater than average can be seen as hub nodes). A statistical analysis was conducted on the relationship (including activation, inhibition, and unspecified) in each system and the relationship among the three systems. The structural characteristics of the regulatory subnetworks were also constructed and analyzed. Furthermore, we used the network topology of the average shortest path length to measure the distance between the signaling molecules in the network. The Wilcoxon rank sum test was conducted to evaluate the significance of the different systems.

### 4.5. Construction of the Disease-Specific NIM Subnetwork

Research on complex diseases by the molecular network level and system angle may be an important means of understanding and treating diseases [88]. With the development of gene expression chip technology, the cost is reduced and samples have been accumulated. The gene expression profile has been widely used as a powerful tool to describe information on gene expression abundance for cells or tissues in particular states, such as disease status or drug disturbance. However, the effective methods of mining gene expression data seem to be a bit overstretched. Therefore, in order to explore how the NIM molecular network has been changed in a particular state, the gene expression profile was a good choice. Considering the complexity of the disease and the cyclic characteristics of the signaling molecules, the transcriptome data of blood samples from the patient, such as the samples gathered from the peripheral blood, whole blood, or peripheral blood mononuclear cells (PBMC), were adopted in the two study cases.

In this study, we take RA and AD as an example to show the application of NIMNT in the prediction of disease targets and the interpretation of disease pathogeneses. We retrieved data from the GEO database (https://www.ncbi.nlm.nih.gov/geo/) to obtain the gene expression profiles of RA and AD. Firstly, we determined the retrieval term to be used by the MeSH (https://www.nlm.nih.gov/mesh/). Then we used the advanced search tools of the GEO database to obtain the AD-related expression datasets, in which we filtered the retrieval results by organism (*Homo sapiens*), study type (expression profiling by array), and blood samples (including peripheral blood, whole blood, and peripheral blood mononuclear cells (PBMC)). Finally, only the studies making a comparative study of the differences between the health control and disease status were chosen in the candidate dataset. The GEO2R tool of GEO was used to obtain the DEGs of the dataset. The genes (*p* < 0.05) were selected as the gene expression profile signature of RA and AD (Tables S20 and S21). 

We took the extended NIM network of the signaling molecules as the background and used the forest algorithm in the Omics integrator software package [89] to extract the RA- and AD-specific NIM subnetwork. The core parameters of the forest algorithm include the dummy edge weight (ω), the edge reliability (β), and the degree penalty (D). They were set to be 3, 6, and 5, respectively, in this study.

### 4.6. Permutation Test

In order to test the reliability of the disease subnetworks, we conducted a permutation test for the disease NIM subnetworks. We downloaded the biomarkers (RA- and AD-related genes) from the MetaCore database as the gold standard (Tables S26 and S27). The permutation test was conducted as follows. For RA- or AD-specific NIM subnetworks, two kinds of nodes were contained: the DEGs and non-DEGs (intermediate nodes). We assumed that the gene numbers contained in these two parts were M1 and M2, respectively. Next, we recorded the numbers of disease-related genes in these two parts as N1 and N2, respectively. For these two parts, we separately conducted permutation tests 1000 times. Then, we counted the times that the number of disease-related genes was greater than N1 or N2, and summed them up as X. The ratio of X/1000 was defined as the *p*-value. Finally, we drew the probability density curve of the number of disease-related genes.

## 5. Conclusions

In this paper, the core dataset of the NIM signaling molecules was collected, and the core and extended networks of NIM signaling molecules were constructed based on the MetaCore database with a manually audited multilayered, multidimensional background network. Through analyzing the characteristics of the core NIM network, we found that this network conformed to the characteristics of a biological network with a small world and a scale-free property, and imbalance characteristics of the signaling transduction between the three systems. To show the application of the NIM molecular network, we conducted two study cases for the prediction of disease targets and the interpretation of disease pathogeneses, respectively. Taking the extended network of the NIM signaling molecules as the background and RA and AD as the study cases, the RA- and AD-specific NIM subnetworks were extracted using the forest algorithm. We examined the reliability of the disease NIM subnetworks using a permutation test. The results showed that the subnetworks were reliable and had a good performance in predicting the disease targets. Our study indicates that the NIM molecular network may facilitate the application of the NIM network in the prediction of disease targets and the interpretation of disease pathogenesis. The NIM network can also be applied to the extraction of a drug action network, explaining the mechanism of the drug and guiding the development of more effective and less toxic drugs. Therefore, research strategies based on NIM networks are very efficient and concise. This is meaningful for the study of the pathogenesis of diseases and the mechanisms of drug action in the context of the NIM network system. 

## Figures and Tables

**Table 1:** The top 10 signaling molecules in the core network.

Gene Symbol	Gene Name	Entrez Gene IDs	Degree	System Category ^1^
*ESR1*	Estrogen receptor 1	2099	98	nervous; endocrine
*PPARG*	Peroxisome proliferator-activated receptor gamma	5468	51	endocrine
*VDR*	Vitamin D receptor	7421	45	endocrine
*HNF4A*	Hepatocyte nuclear factor 4 alpha	3172	43	nervous; endocrine
*ESR2*	Estrogen receptor 2	2100	43	endocrine
*PGR*	Progesterone receptor	5241	39	endocrine
l-Glutamic acid	l-Glutamic acid	/	27	nervous
Glycine	Glycine	/	27	nervous
Dopamine	Dopamine	/	27	nervous

^1^ The system that the signaling molecules belong to.

**Table 2:** The top 4 hub genes in each category in the rheumatoid arthritis (RA)-specific NIM subnetwork based on degree.

Gene Category	Gene Symbol	Gene Name	Entrez Gene ID	Degree
Differentially expressed ^1^ and RA-related genes	*CREB1*	cAMP-responsive element-binding protein 1	1385	9
	*TNFSF10*	TNF superfamily member 10	8743	4
	*CD4*	CD4 molecule	920	3
	*TNFSF12*	TNF superfamily member 12	8742	3
Non-differentially expressed and RA-related genes	*ESR1*	Estrogen receptor 1	2099	95
	*VDR*	Vitamin D receptor	7421	42
	*PPARG*	Peroxisome proliferator-activated receptor gamma	5468	22
	*ESR2*	Estrogen receptor 2	2100	14
Differentially expressed and RA-unrelated genes	*GSK3B*	Glycogen synthase kinase 3 beta	2932	8
	*SMARCA4*	SWI/SNF-related, matrix-associated, actin-dependent Regulator of chromatin, subfamily a, member 4	6597	7
	*PSMD7*	Proteasome 26S subunit, non-ATPase 7	5713	6
	*TGFBR1*	Transforming growth factor beta receptor 1	7046	4
Non-differentially expressed and RA-unrelated genes	*HNF4A*	Hepatocyte nuclear factor 4 alpha	3172	32
	*PGR*	Progesterone receptor	5241	27
	*RXRA*	Retinoid X receptor alpha	6256	23
	*ESRRA*	Estrogen-related receptor alpha	2101	18

^1^ Gene expression profile dataset of RA is from GSE15573 [33] in the GEO database.

## Supplementary Materials

The supplementary materials are available online. Figure S1: The probability density curve of the average shortest path length; Figure S2: The Wilcoxon rank sum test for the average shortest path length; Figure S3: The tissue specificity analysis of the signaling molecules; Table S1: The retrieval words and corresponding results for the NIM signaling molecules; Table S2: The signaling molecules belonging to the nervous system (genes); Table S3: The signaling molecules belonging to the nervous system (compounds); Table S4: The signaling molecules belonging to the endocrine system (genes); Table S5: The signaling molecules belonging to the endocrine system (compounds); Table S6: The signaling molecules belonging to the immune system (genes); Table S7: The tissue specificity analysis of the signaling molecules belonging to the nervous system; Table S8: The tissue specificity analysis of the signaling molecules belonging to the endocrine system; Table S9: The tissue specificity analysis of the signaling molecules belonging to the immune system; Table S10: The network topology parameter data for the core network of the NIM signaling molecules; Table S11: The network topology parameter data for the internal regulatory network of NSSM; Table S12: The network topology parameter data for the regulatory network from NSSM to ESSM; Table S13: The network topology parameter data for the regulatory network from NSSM to ISSM; Table S14: The network topology parameter data for the regulatory network from ESSM to NSSM; Table S15: The network topology parameter data for the internal regulatory network of ESSM; Table S16: The network topology parameter data for the regulatory network from ESSM to ISSM; Table S17: The network topology parameter data for the regulatory network from ISSM to NSSM; Table S18: The network topology parameter data for the regulatory network from ISSM to ESSM; Table S19: The network topology parameter data for the internal regulatory network of ISSM; Table S20: The gene expression profile signature of RA; Table S21: The gene expression profile signature of AD; Table S22: The network topology parameter data for the RA-specific NIM subnetwork; Table S23: The network topology parameter data for the AD-specific NIM subnetwork; Table S24: The edge data of the RA-specific NIM subnetwork; Table S25: The edge data of the AD-specific NIM subnetwork; Table S26: The biomarker dataset of RA downloaded from the MetaCore database; Table S27: The biomarker dataset of AD downloaded from the MetaCore database; Table S28: The extended network of NIM signaling molecules.
