# Supplementary material for: Knowledge-Based Neuroendocrine Immunomodulation (NIM) Molecular Network Construction and Its Application
Source: Molecules. 2018 May 30;23(6):1312. doi: 10.3390/molecules23061312 (PMC6099962; doi:10.3390/molecules23061312)
Supplement: Supplementary file 1 [file molecules-23-01312-s001.zip › Table S1 Retrieval words and corresponding results for NIM signaling molecules.docx]

**Supplementary Table S1.** Retrieval words and corresponding results for NIM signaling molecules.

| **System category** | **Keyword** | **Gene ontology** | **Chemical ontology** | **Unique total number^*^** |
| --- | --- | --- | --- | --- |
| Nervous system | neurotransmitter | neurotransmitter receptor activity (GO:0030594) | neurotransmitter (CHEBI: 65512) | 29+21 |
|  | neuropeptide | neuropeptide hormone activity (GO: 0005184) | / | 61 |
|  |  | neuropeptide receptor activity (GO: 0008188) | / |  |
| Endocrine system | hormone | hormone activity (GO: 0005179) | hormone (CHEBI: 24621) | 187+47 |
|  |  | hormone receptor activity (GO: 0004903) | / |  |
|  |  | luteinizing hormone receptor activity (GO: 0004964) | / |  |
|  |  | follicle-stimulating hormone receptor activity (GO: 0004963) | / |  |
|  |  | gonadotropin-releasing hormone receptor activity (GO: 0004968) | / |  |
|  |  | thyroid hormone receptor activity (GO: 0004887) | / |  |
|  |  | adipokinetic hormone receptor activity (GO: 0097003) | / |  |
|  |  | melanocyte-stimulating hormone receptor activity (GO: 0004980) | / |  |
|  |  | parathyroid hormone receptor activity (GO: 0004991) | / |  |
|  |  | thyrotropin-releasing hormone receptor activity (GO: 0004997) | / |  |
|  |  | thyroid-stimulating hormone receptor activity (GO: 0004996) | / |  |
|  |  | ecdysis-triggering hormone receptor activity (GO: 0042654) | / |  |
|  |  | steroid hormone receptor activity (GO: 0003707) | / |  |
|  |  | protein-hormone receptor activity (GO: 0016500) | / |  |
|  |  | growth hormone-releasing hormone receptor activity (GO: 0016520) | / |  |
|  |  | choriogonadotropin hormone receptor activity (GO: 0035472) | / |  |
|  |  | anti-Mullerian hormone receptor activity (GO: 1990272) | / |  |
|  |  | diuretic hormone receptor activity (GO: 0008036) | / |  |
| Immune system | cytokine | cytokine activity (GO:0005125) | / | 302 |
|  |  | cytokine receptor activity (GO:0004896) | / |  |
| Total number | | | | 611 |

*genes number + compounds number.
