# Supplementary figures and images for: Knowledge-Based Neuroendocrine Immunomodulation (NIM) Molecular Network Construction and Its Application
Source: Molecules. 2018 May 30;23(6):1312. doi: 10.3390/molecules23061312 (PMC6099962; doi:10.3390/molecules23061312)

**(A)**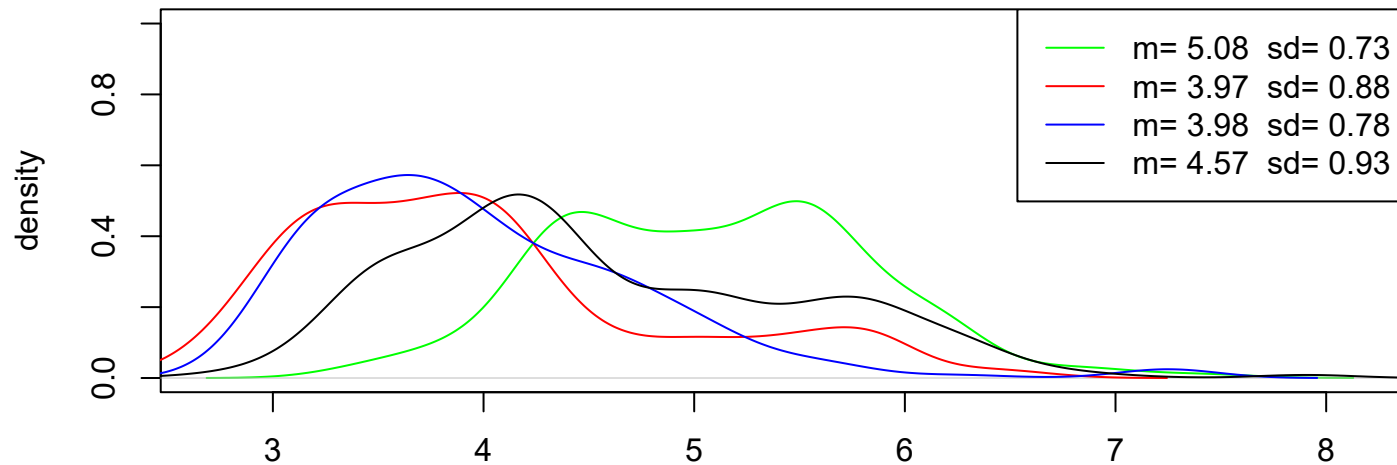**(B)**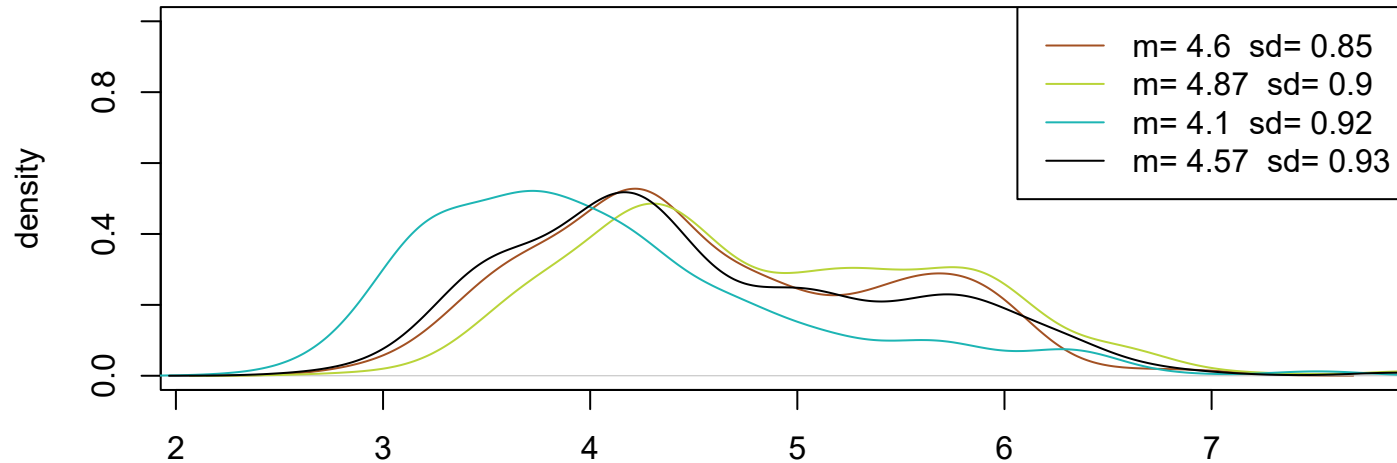

Supplement: Supplementary file 1 [file molecules-23-01312-s001.zip › Figure S1.pdf]

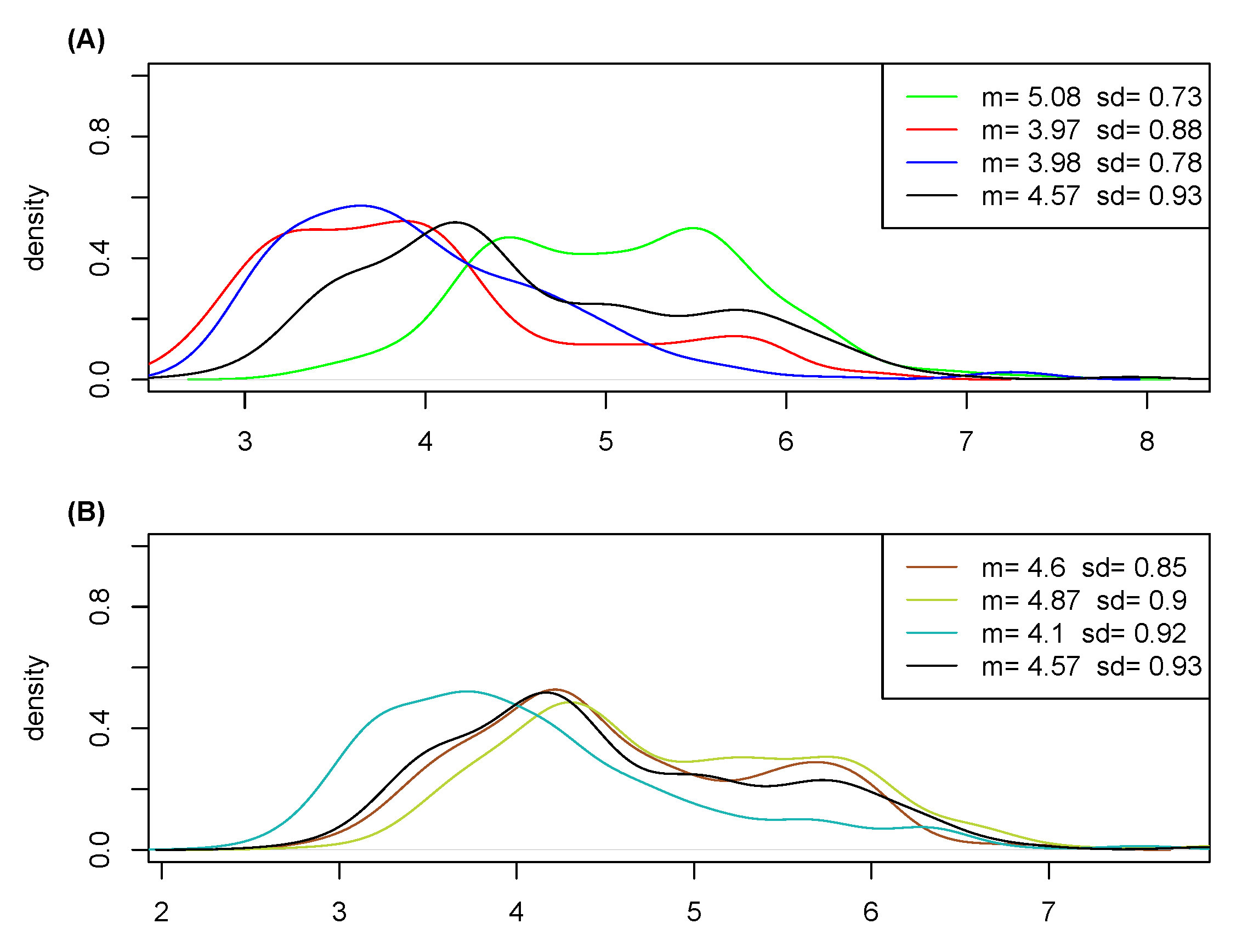

Supplement: Supplementary file 1 [file molecules-23-01312-s001.zip › Figure S1.tiff]

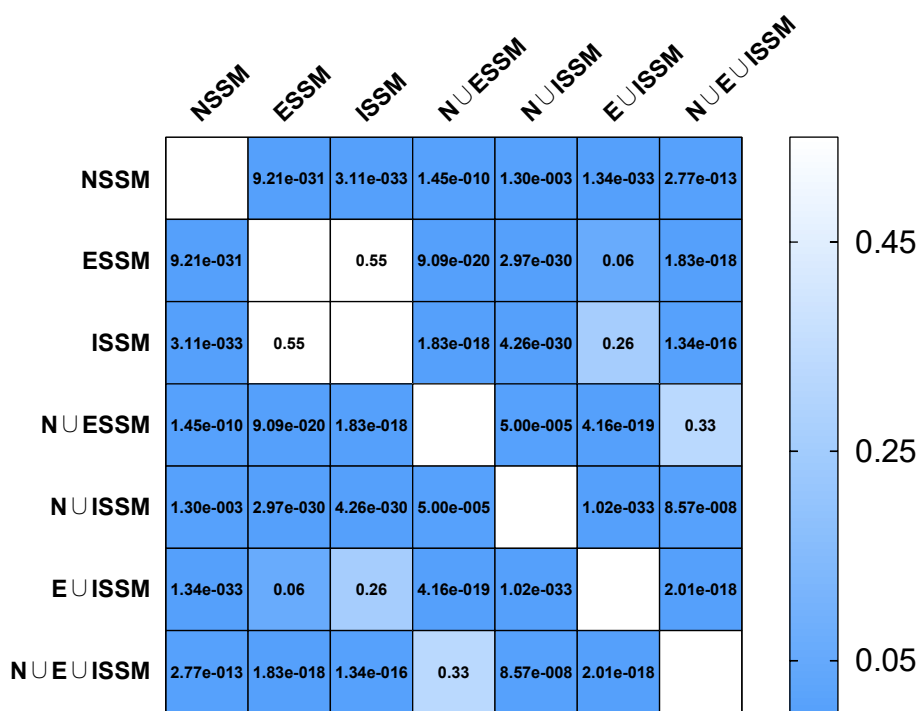

Supplement: Supplementary file 1 [file molecules-23-01312-s001.zip › Figure S2.pdf]

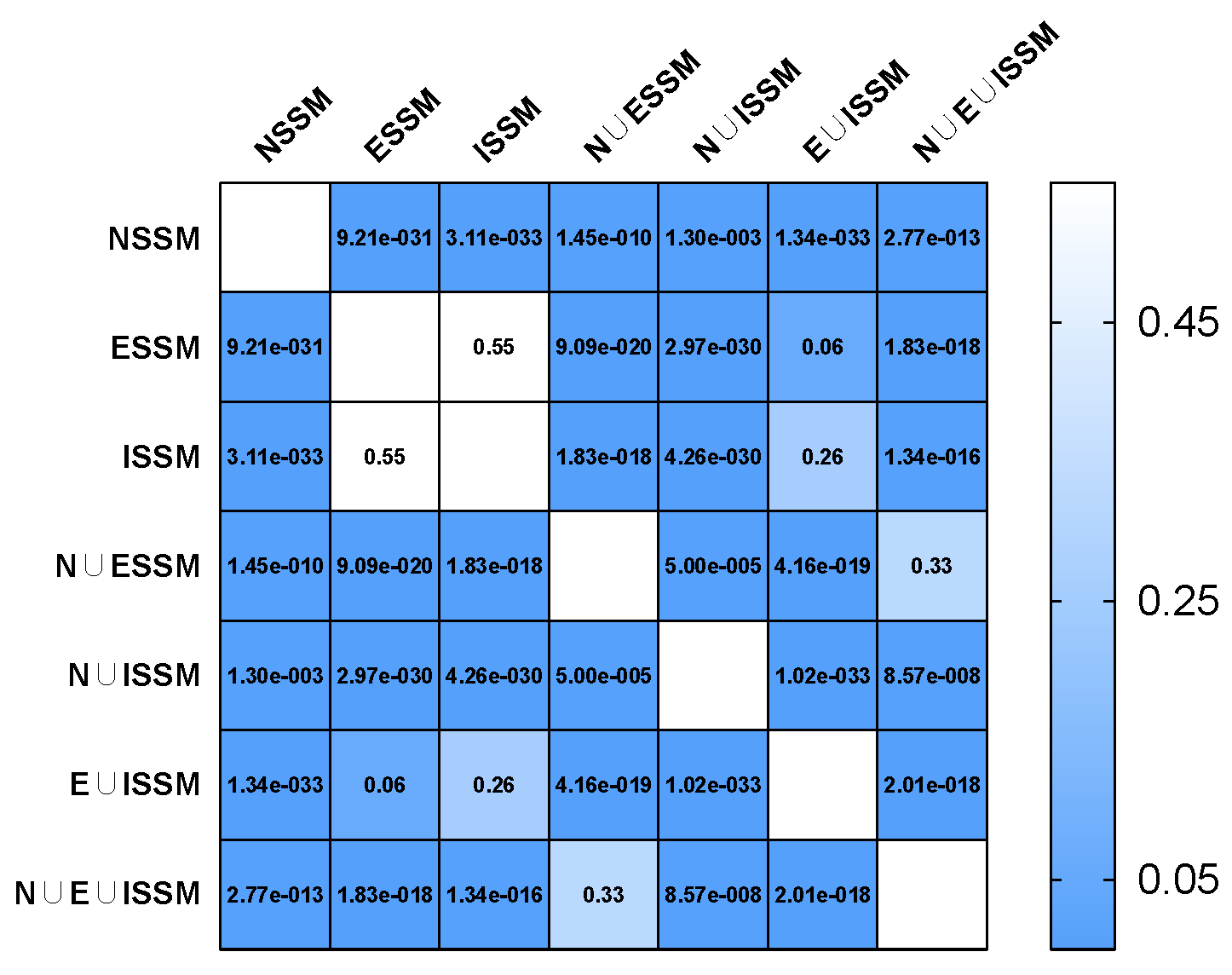

Supplement: Supplementary file 1 [file molecules-23-01312-s001.zip › Figure S2.tiff]

**(A)**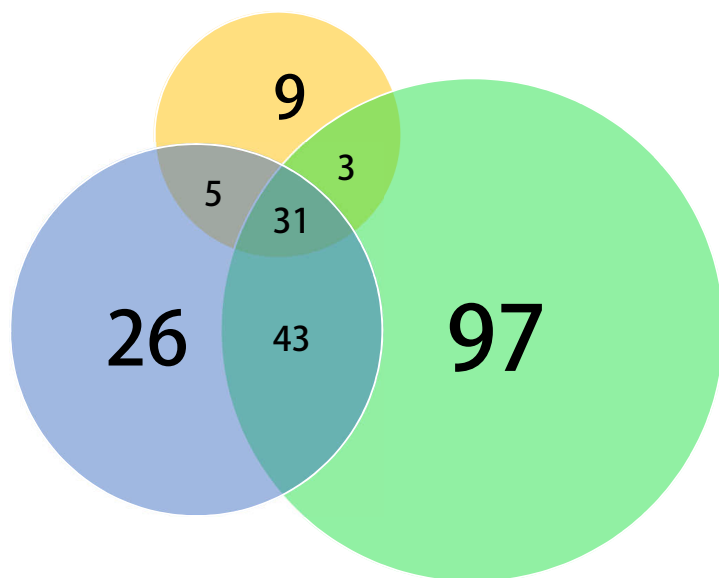**(B)**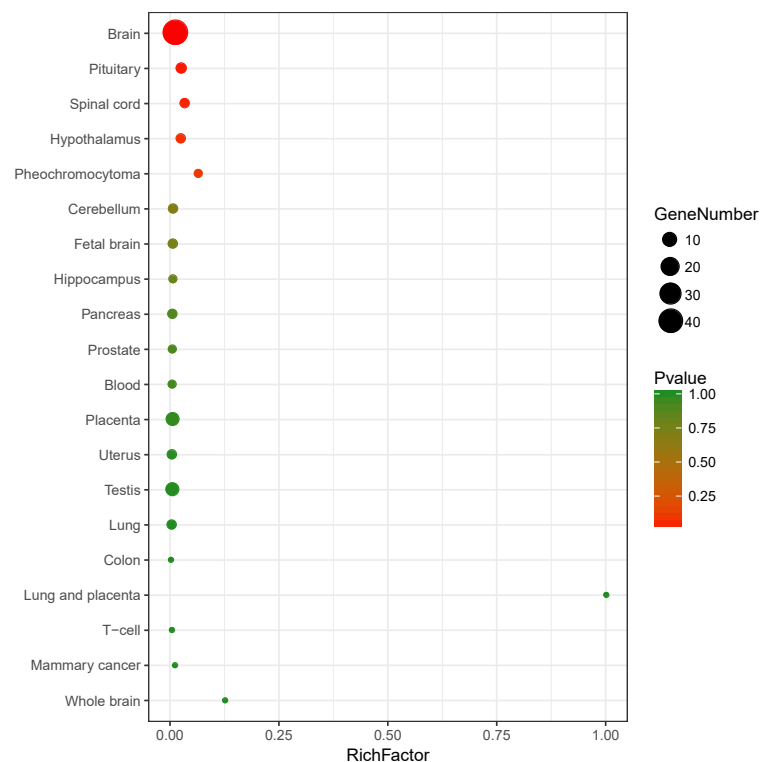**(C)**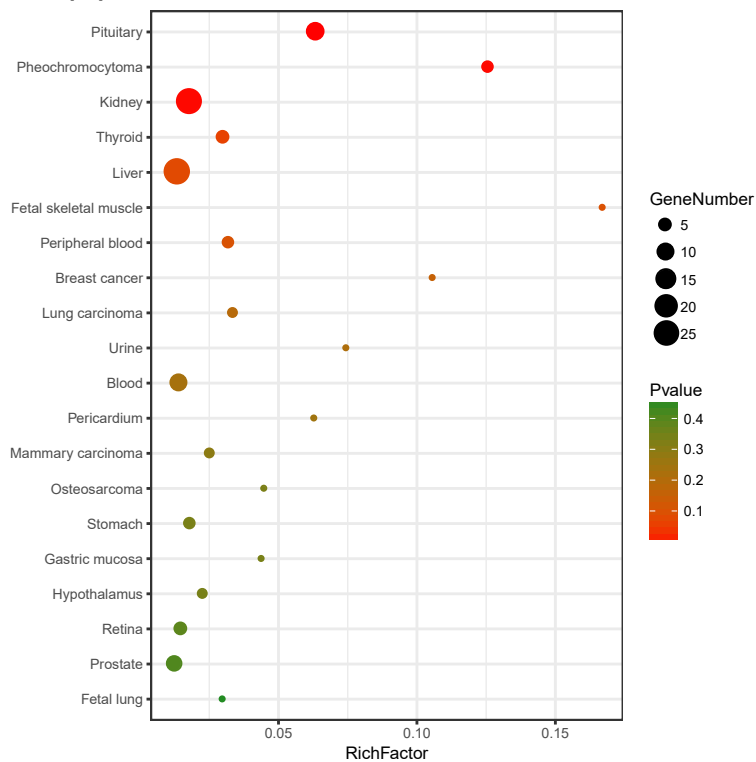**(D)**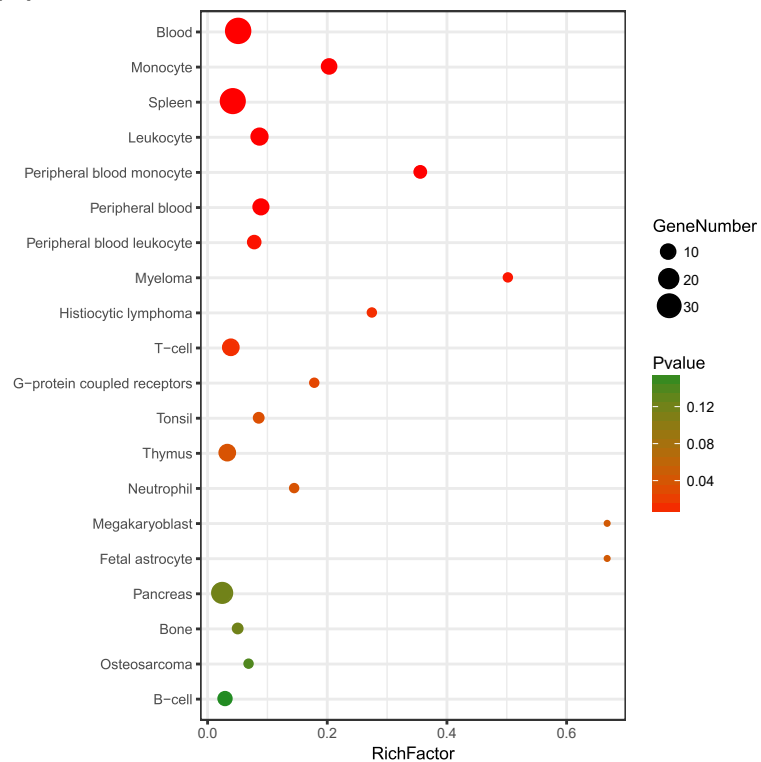

Supplement: Supplementary file 1 [file molecules-23-01312-s001.zip › Figure S3.pdf]

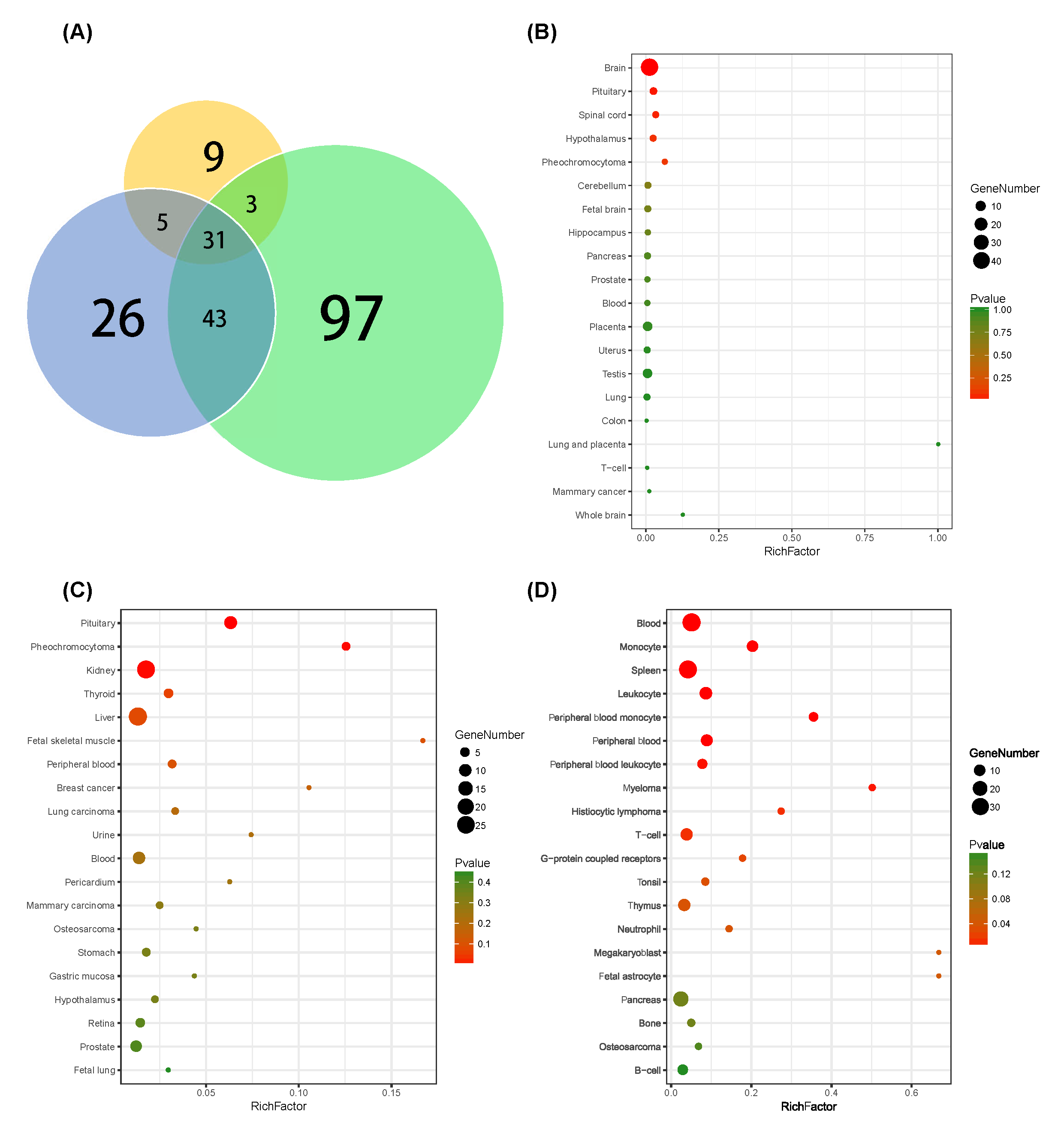

Supplement: Supplementary file 1 [file molecules-23-01312-s001.zip › Figure S3.tiff]
